# Supplementary material for: Tele-Exercise for Fitness: Physical and Psychological Outcomes in Athletes and Non-Athletes’ Trainees
Source: Healthcare (Basel). 2025 Feb 7;13(4):354. doi: 10.3390/healthcare13040354 (PMC11855133; doi:10.3390/healthcare13040354)
Supplement: Supplementary file 1 [file healthcare-13-00354-s001.zip › healthcare-3429158-supplementary.pdf]

healthcare

MDPI

Supplementary materials of the Article ***Tele-Exercise for Fitness: Physical and Psychological Outcomes in Athletes and Non-Athletes' Trainees***

Dropout data

Dropout rates are reported in Table S1, 334 accesses to TELEexe4all platform were observed, the procedure was reported in the flowchart in figure 1. We considered only if the 8-weeks programs and the survey was 100% completed. Subjects who have repeated the programs were excluded. In Hoerger et al. 2010, was reported that: i) in short and less demanding studies (e.g., brief questionnaires or surveys), the dropout rate is usually low, around 5-10%; 2) in the Long-term or demanding studies (such as interventions requiring prolonged commitment), the dropout rate can increase to 20-30% or even higher; 3) in clinical or intervention studies (especially those involving lifestyle changes, medical treatments, or significant alterations) it can range from 20-50%; 4) the Online studies often have higher dropout rates compared to in-person studies, sometime es exceeding 30%, especially if there is no direct contact with participants or adequate incentives. In Galesic et al. 2006 we found that "In general invitation web survey, which mostly recruit respondents through web-based advertisement, dropout ratee may be as high as 80 percent". In this study, the athletes group reported a higher dropout in PSY survey (58%) on respect athletes' group (27%) (see Table S1).

Table S1. Dropout rate

Groups	N	PSY survey (23 Items)		Fitness test (4 tests)	
	Pre	Post	%Dropout	Post	%Dropout
**Athletes**	161	67	58% (94/161)	-	-
**Non-Athletes**	173	126	27% (47/173)	92	47% (81/173)
**All**	334	193	42% (141/334)	-	-

PSY survey: physiological survey; Fitness test was administered to non-athletes only.

Participants' characteristics for each subgroup

Below in Table S2 the characteristics of the participants in the study are shown, presented all together and divided into each subgroup.

Table S2. Participants' characteristics divided for subgroups

Group	Age (years)	Ath./Non-ath.	Males/Females	SY/AS	Weight (kg)	Height (cm)
**All (193)**	36.64±11.87	67/126	77/116	123/70	71.30±15.00	169.67±9.74
**Athletes (67)**	36.36±8.95	67/0	34/33	56/11	74.81±16.67	168.83±9.46
**Non-athletes (126)**	36.79±13.20	0/126	43/83	67/59	69.74±14.01	169.93±10.93
**Males (77)**	35.51±10.95	34/43	77/0	50/27	78.53±14.2	176.05±10.35
**Females (116)**	38.31±12.68	83/33	0/116	73/43	66.78±13.35	165.32±6.34
**SY (123)**	39.79±14.19	56/67	50/73	123/0	71.74±14.17	169.10±9.63
**AS (70)**	32.90±5.21	11/59	27/43	0/70	71.07±17.38	171.93±9.98
**Young (24)**	23.98±5.82	10/14	15/9	20/4	70.38±11.05	174.04±13.45
**Adult (142)**	34.98±4.36	47/95	52/90	76/66	70.36±15.54	168.61±8.47
**Middle age (18)**	49.78±4.02	10/8	8/10	18/0	75.94±16.87	170.22±9.28
**Elderly (9)**	77.33±5.05	0/9	2/7	9/0	78.22±10.08	167.22±8.5

Age, Weight and Height are reported as means ± standard deviation; Gender and Mode are reported as numerosity; Ath. = Athletes; AS = Asynchronous; SY = Synchronous.

References

1. Hoerger, M. Participant dropout as a function of survey length in Internet-mediated university studies: Implications for study design and voluntary participation in psychological research. *Cyberpsychol Behav Soc Netw* **2010**, 13(6), 697-700. doi: 10.1089/cyber.2009.0445.
2. Galesic, M. (2006). Dropouts on the web: Effects of interest and burden experienced during an online survey. *J Off Stat* **2006**, 22(2), 313.
